# MEMS Microphone Array Sensor for Air-Coupled Impact-Echo

**DOI:** 10.3390/s150714932

**Published:** 2015-06-25

**Authors:** Robin Groschup, Christian U. Grosse

**Affiliations:** Technische Universität München (TUM), Chair of Non-destructive Testing, Baumbachstr. 7, 81245 Munich, Germany; E-Mail: grosse@tum.de

**Keywords:** nondestructive testing of concrete, Impact-Echo, air coupled sensing, array techniques, MEMS microphones

## Abstract

Impact-Echo (IE) is a nondestructive testing technique for plate like concrete structures. We propose a new sensor concept for air-coupled IE measurements. By using an array of MEMS (micro-electro-mechanical system) microphones, instead of a single receiver, several operational advantages compared to conventional sensing strategies in IE are achieved. The MEMS microphone array sensor is cost effective, less sensitive to undesired effects like acoustic noise and has an optimized sensitivity for signals that need to be extracted for IE data interpretation. The proposed sensing strategy is justified with findings from numerical simulations, showing that the IE resonance in plate like structures causes coherent surface displacements on the specimen under test in an area around the impact location. Therefore, by placing several MEMS microphones on a sensor array board, the IE resonance is easier to be identified in the recorded spectra than with single point microphones or contact type transducers. A comparative measurement between the array sensor, a conventional accelerometer and a measurement microphone clearly shows the suitability of MEMS type microphones and the advantages of using these microphones in an array arrangement for IE. The MEMS microphone array will make air-coupled IE measurements faster and more reliable.

## 1. Introduction

Impact-Echo (IE) is a nondestructive method that can be used to identify geometric dimensions and internal defects of concrete structures [1,2,3]. A main advantage of IE is that only one-sided access is necessary. Recent developments have shown that IE has high potential in certain measuring tasks where other methods come to their limits. These tasks range from backfill detection of tunnel linings [4] to the detection of delaminations in concrete [5,6,7,8]. During an IE test stress waves are induced by a mechanical impact (e.g., impact hammer or steel ball drop) on the surface of the tested specimen. The excited stress waves are normally measured at a single sensor location in the vicinity of the impact point. In terms of ultrasonics, the frequencies relevant for the IE method can be understood as low frequency waves. This brings advantages since concrete is a highly attenuative medium for ultrasonic waves and low frequency waves suffer less attenuation than high frequency waves. In the usual IE processing scheme the sensor recordings are transformed from time domain to frequency domain. In plate like structures the resulting spectra show a frequency peak that can be related to the plate thickness via the relation:

$$f_{r}=\frac{\text{β}\;v_{P}}{2d}$$

where *f_r_*: maximum frequency in recorded signal; *v*_p_: P-wave velocity; *d*: plate thickness; and β: dimensionless correction factor, typical value for concrete 0.96. *f_r_* is often referred to as thickness resonance (or IE resonance) and can be theoretically explained as the frequency of the S_1_ Lamb mode with zero group velocity (ZGV) [9]. With the correct adjustment of the contact time and contact area of the impact device, this wave type has a high excitability in plate like structures. The stress waves on the surface of a concrete specimen also induce acoustic waves in the surrounding air. This enables the use of air coupled receivers such as conventional microphones as sensors for air-coupled IE measurements [10,11,12]. Air-coupling allows for fast measurement speeds in a scanning measurement mode since no direct coupling of a sensing element and the tested surface is necessary [13].

However, an inherent problem to the IE method is the correct extraction of the ZGV-S_1_ Lamb wave. In real world structures, the IE resonance frequency cannot always be clearly identified due to the superposition of other wave types such as boundary reflections of surface waves or higher modes of body vibrations [14,15]. Several methods have been proposed to enhance the signal of the IE resonance to facilitate further signal interpretation, ranging from improved processing schemes [16,17] to the usage of multiple hammer impacts in the surrounding of a single sensing location [4].

The latter approach can be inverted due to the reciprocity law in wave propagation that allows the exchange of receiver and source positions. We propose a novel IE sensor consisting of multiple microphones in an array arrangement. The sensor concept combines operational advantages of air-coupled sensors with increased sensitivity to the desired IE resonance signal. Besides the enhanced sensitivity, the new sensing approach has other significant advantages like less sensitivity to the acoustic noise of the direct impact and ambient acoustic noise. Such effects can be great obstacles for implementing air-coupled IE as a routine technique in nondestructive testing of civil engineering structures.

The article is organized as follows: First, we emphasize the nature of the airwave radiation pattern of the ZGV-S_1_ Lamb mode with the help of numerical simulations. Later, we present the implementation of a prototype sensor device that consists of an array arrangement of MEMS (micro-electro-mechanical system) microphones optimized for IE testing. The prototype is tested in a typical IE measurement on a concrete wall.

## 2. Numerical Simulation of IE Test

**Figure 1:**
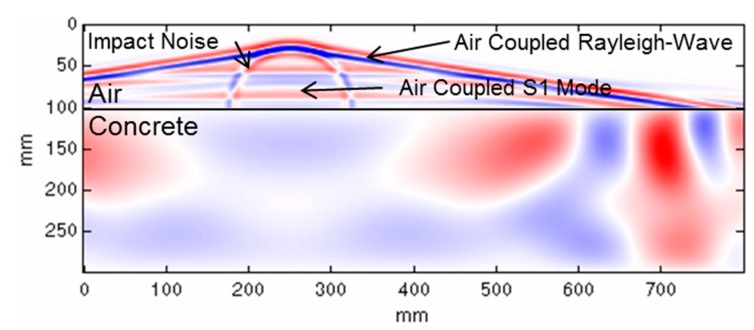
Snapshot of a simulation of stress waves inside concrete and radiated acoustic waves [18].

The stress waves inside a concrete plate after an impact and the radiated waves in the surrounding air can be visualized by a 2D finite element simulation (Figure 1, [18]).

**Figure 2:**
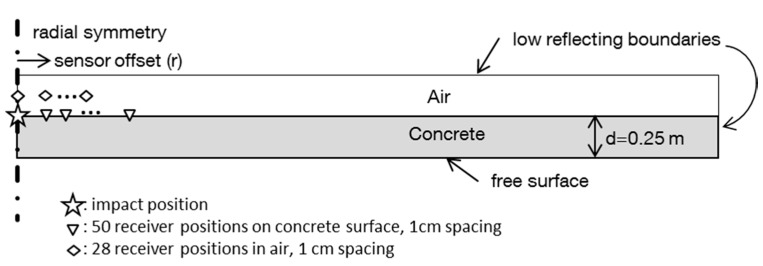
Setup for the numerical model.

Our goal is the design of an acoustic sensor with optimum sensitivity to the air coupled ZGV-S_1_ mode. So we are going to examine in detail the pressure changes in the air that arise from the surface movement of the concrete element caused by the ZGV-S_1_ Lamb wave. This wave is visible in the air as plane wave fronts. This fact implies that the wave arrives in phase along a certain aperture surrounding the impact position. To further investigate the behavior of this peculiar waveform, we rely on a more realistic simulation. We adopt a simulation approach proposed by Castaings *et al.* [19] and applied to IE by Baggens and Ryden [20]. The propagation of stress waves and the radiation of acoustic waves are studied by a finite element simulation in the frequency domain. The model consists of a plate of concrete (thickness 0.25 m) with an adjacent layer of air at the topside (Figure 2). The modeling was performed with a cylindrical symmetry (impact position in the center of symmetry) and a low reflecting boundary at the outer edges of the model. A free boundary was set at the lower surface of the model. A Gaussian monopuls with center frequency 15 kHz was used as the excitation source. Typical values were chosen for the elastic properties of concrete (Young’s modulus 36 GPa, density 2300 kg/m^3^, Poisson’s ratio 0.2).

**Figure 3:**
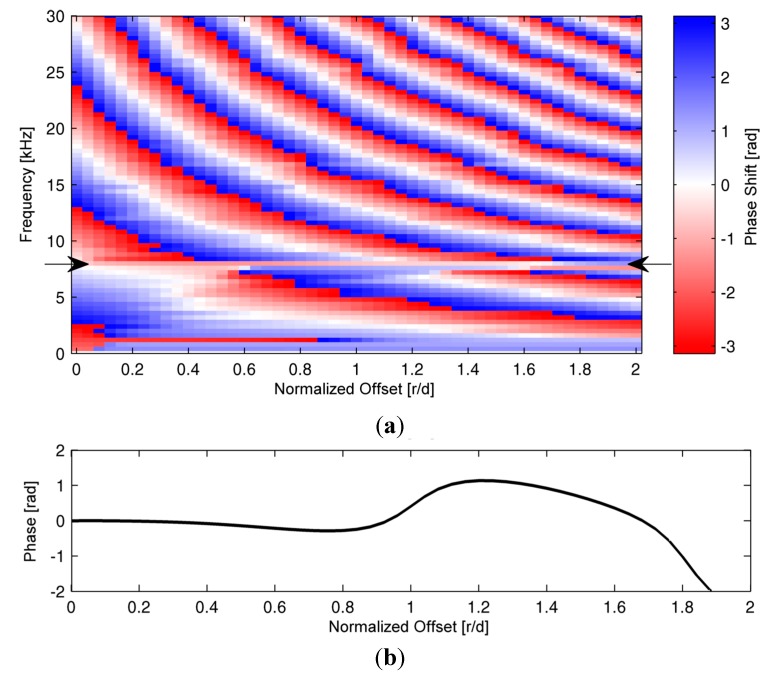
(**a**) Phase spectra of displacement recordings on the surface of the simulated concrete plate. The arrows indicate the frequency of the IE resonance; (**b**) Graph display of phases of displacement recordings at the IE resonance.

For the presentation of simulation results we put emphasis on the surface displacements in a region around the impact position. Figure 3 shows the phase behavior of displacements measured by receivers placed on a line on the concrete surface. Most of the wave energy shows continuous phase shifts along the offset axis. This is due to the propagation of different wave modes along the sensor line. Only at the frequency of the ZGV-S_1_ Lamb mode—the so-called IE resonance frequency—wave phases remain constant over a considerable length (see arrows in Figure 3). In our simulation example, this frequency lies at 8 kHz and thus coincides well with the value predicted by the formula mentioned in Section 1 (for the chosen elastic parameters the concrete has a P-wave velocity of 4170 m/s).

**Figure 4:**
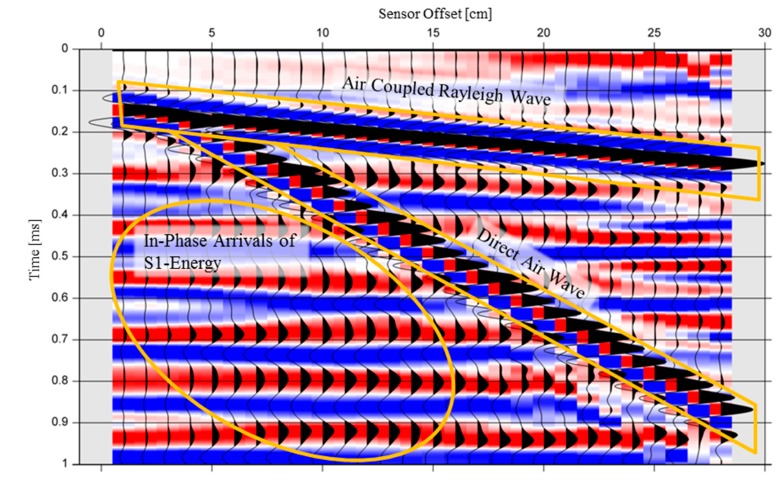
Recordings of pressure changes in the air in the simulation model. For better visualization, each trace is individually scaled to its maximum value.

Figure 3b gives a detailed view of the phase behavior at the IE resonance frequency as a function of sensor distance to impact position normalized to the plate thickness. It becomes clear that the signal of the ZGV-S_1_ Lamb wave shows constant phases in in a vicinity of the impact location up to a distance that is comparable to the plate’s thickness. Therefore, the wavefield radiated into the air is coherent in this region and consists of plane wave fronts. This effect can also clearly be seen by a visualization of pressure recordings above the impact location (Figure 4).

## 3. Design of a MEMS Based Microphone Array

**Figure 5:**
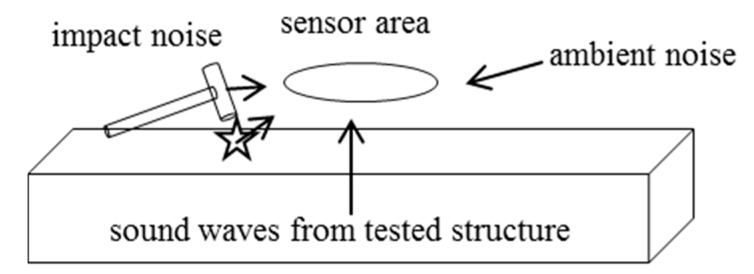
Sound sources acting on sensors in air-coupled Impact-Echo (IE).

For optimizing the sensitivity of an air-coupled sensor for IE purposes, two approaches can be considered. Either the plane wave fronts emerging from the air-coupled ZGV-S_1_ mode could be physically focused towards a single microphone [21] or the air-coupled waves could be sensed by an array arrangement of microphones within the zone where the ZGV-S_1_ mode produces in-phase pressure changes. We focus on the latter approach since it brings a number of operational advantages. Based on the findings from the simulation, IE measurements can be effectively performed by placing several receivers in a region around the impact location that stretches approximately up to the plate thickness (the first nodal point of the ZGV-S_1_ Lamb mode). All of these sensors would receive the same waveform information and therefore the summed output of these receivers would be more sensitive to the displacements of the plate surface due to the thickness resonance than the output of a single receiver alone. This fact comes in quite handy when using air-coupled sensing with microphones since such sensors will always pick up unwanted energy from ambient noise or noise from the direct impact (Figure 5).

The desired signal in IE consists of plane wave fronts emerging from the surface of the element under test, whereas ambient noise or direct noise from the impact comes from the sides. In practice, the noise from the impact device can be a huge handicap since it comprises not only a short duration transient wave. It can also contain longer lasting oscillations due to vibrations of the impact device.

**Figure 6:**
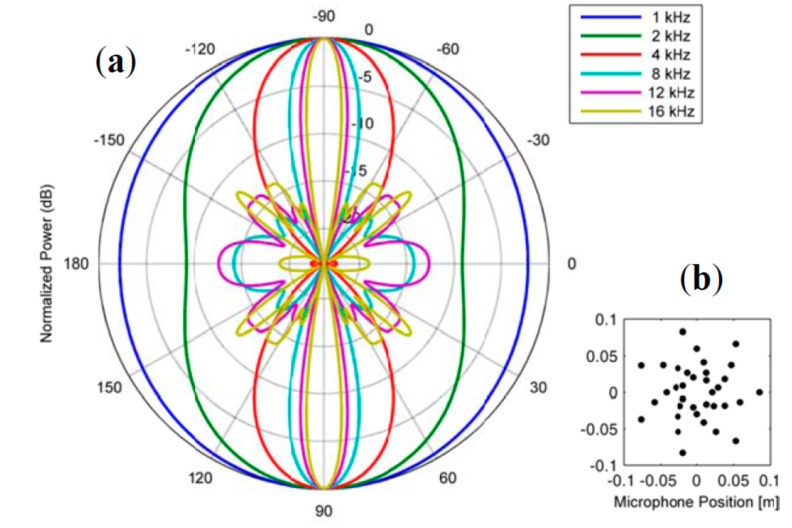
(**a**) Directional sensitivity pattern for a microphone array consisting of 35 single microphones, spatial arrangement shown in (**b**).

The employment of several microphones as a single sensing element can be understood as an optimization of the directivity pattern of the combined sensing element. When defining the actual arrangement of microphones in an array, one has to consider the frequency dependence of the directional sensitivity (also termed polar pattern). To suppress side lobes, uniform distances between single microphones have to be avoided [22,23]. Due to the customizable and steerable directivity such array arrangements are commonly used in the field of technical acoustics for source localization tasks [23]. We adopted such a design to fit to the requirements for IE testing of typical plate structures. Figure 6 shows the polar pattern of the proposed microphone array arrangement consisting of 35 single microphones.

Whereas each single microphone has omnidirectional sensitivity, an array arrangement of such microphones has its greatest sensitivity for wave fronts that are parallel to the plane of the array (*i.e*., having directions of ±90°). In perpendicular directions, the sensitivity is effectively decreased by at least 8 dB for frequencies greater than 2 kHz. This behavior is very desirable since it would lead to an attenuation of unwanted acoustic signals in the final array output. The 180° symmetry can be avoided by back baffling the array with a suitable sound insulating material to avoid sound incident from the back of the array. This design was chosen because it uses a manageable number of single microphones and has a good directional response for frequencies relevant in IE testing. With the proposed dimensions of the complete array and distances between single microphone elements, a good tradeoff between lateral resolution—which will be limited by the overall diameter of the array—and optimal directivity for frequencies that are of interest for typical IE measurement tasks can be achieved.

**Figure 7:**
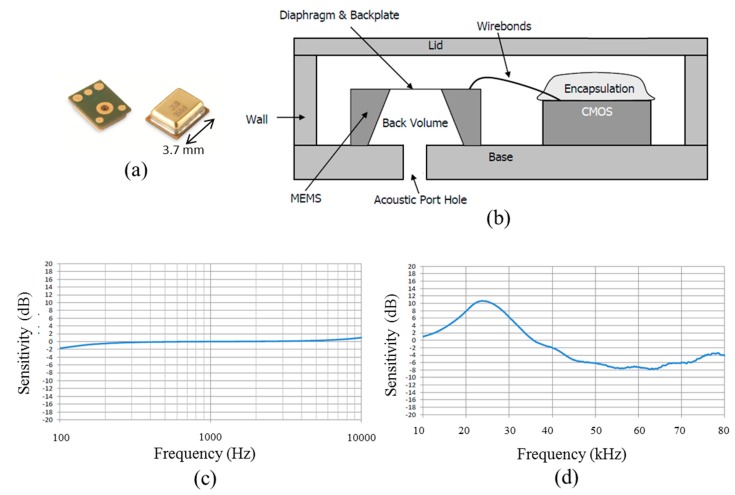
(**a**) Single Micro-Electro-Mechanical Sensor (MEMS) microphone; (**b**) schematic diagram of MEMS microphone; (**c**) frequency response curve for the audio range; and (**d**) frequency response curve for ultrasound of the used MEMS microphone. Reproduced with permission from [25].

Typical civil engineering structures, such as plates made of concrete ranging in thickness from several centimeters to around one meter, will show IE resonance frequencies of 2–20 kHz. Thus, microphones for conventional audio purposes are principally suited as IE sensors. When the output of several microphones has to be combined, the sensor characteristics like sensitivity or frequency and phase response should be as similar as possible among all single microphones. Due to the manufacturing process MEMS type microphones fulfill this requirement better than electret based microphone capsules. Ham and Popovics recently showed the suitability of MEMS type microphones for NDT of concrete [24]. For a prototype array, we chose a model of MEMS based microphones similar to the one in the mentioned study (manufacturer: Knowles, type: SPU0410LR5H-QB, Figure 7a,b) described in Table 1 [25]. According to its datasheet, the microphone has a flat frequency response up to 10 kHz and a pronounced resonance peak between 20 kHz and 30 kHz increasing the sensitivity by approximately 11 dB. However, this behavior is no restriction for practical measurements since in the conventional interpretation scheme of IE data only the identification of the positions of frequency peaks is relevant and not the absolute quantification of pressure changes caused by surface displacements.

**Table 1:** Properties of used MEMS microphones [25].

Parameter	Conditions	Minimum	Type	Maximum	Units
Supply Voltage		1.5		3.6	V
Supply Current			120	160	A
Sensitivity	94 dB SPL @ 1 kHz	−41	−38	−35	dBV/Pa
Signal to Noise Ratio	94 dB SPL @ 1 kHz, A-weighted		63		dB(A)
Total Harmonic Distortio	94 dB SPL @ 1 kHz, S = Typ, Rload > 3 kΩ		0.15	0.2	%
Acoustic Overload Point	10% THD @ 1 kHz, S = Typ, VDD = 3.6 V, Rload > 3 kΩ	116	118		dB SPL
DC Output	VDD = 1.5 V		0.73		V
Output Impedance	@ 1 kHz			400	Ω
Directivity			Omnidirectional		
Polarity	Increasing sound pressure		Increasing output voltage		

**Figure 8:**
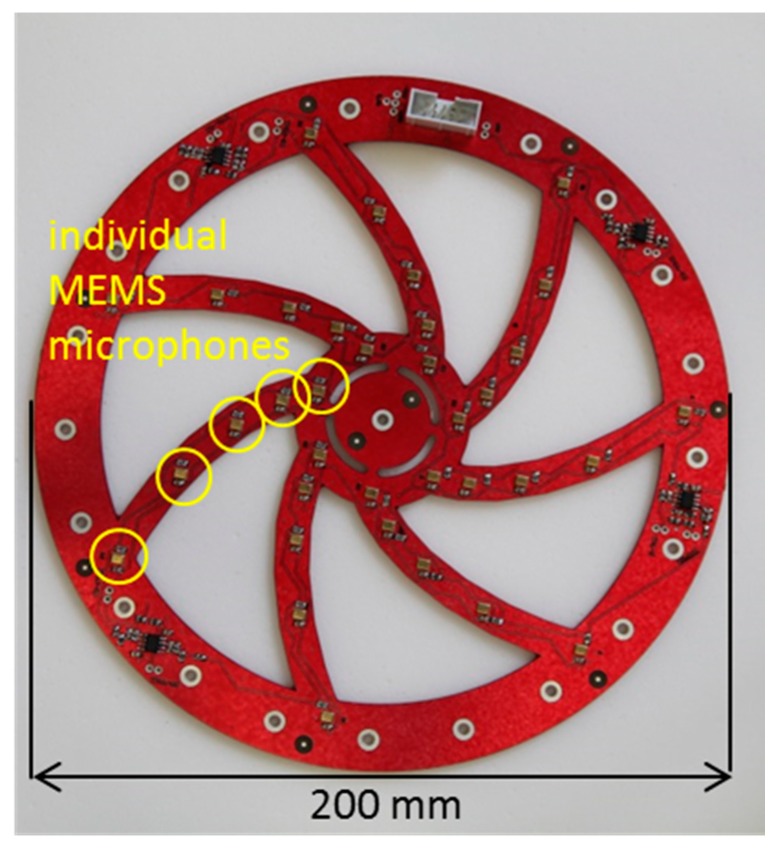
Photograph of the printed circuit board with 35 single MEMS microphones and additional circuitry.

MEMS microphones need additional circuitry for power supply and signal conditioning. These features should not increase the noise level of the single microphones. We employed ultralow noise operational amplifiers to sum up the signals of the individual microphones and to bring the output signal to a level that can be easily measured with a digital oscilloscope or any other conventional recording system. A printed circuit board (PCB) carrying the individual MEMS microphones and the additional circuitry (power supply and preamplifier) was designed. Material on the PCB not needed for supporting individual parts or conducting paths has been removed in order to not influence the sound propagation and to minimize the effects of the supporting structure to the frequency response (Figure 8). In this way, flexibility concerning the use of different back baffling materials or stacking several of these sensor arrays can be maintained.

## 4. Test Measurement with the MEMS Microphone Array

**Figure 9:**
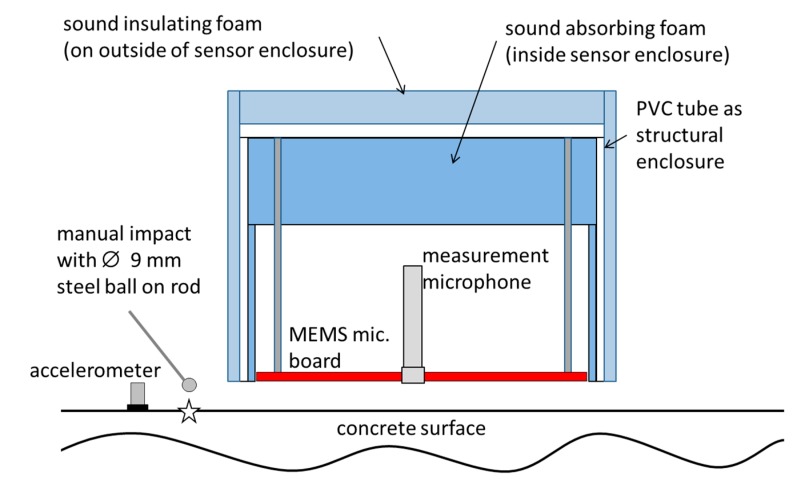
Schematic of the setup for the test measurement.

To make optimal use of the directional characteristics, a specialized enclosure was constructed for the microphone array (Figure 9). The enclosure was designed to keep away sound incident from the backside of the array. As shown in Section 3, such directions of incidence would not be effectively damped. For preventing sound reflections inside the enclosure open-pored sound absorbing foam was applied to the inner side. The enclosure also carried a conventional electret based measurement microphone (sensitivity 50 mV/Pa) in the center of the array arrangement for comparison measurements.

With this setup, an IE measurement was performed on a concrete building wall (thickness 0.2 m ± 0.005 m). The exact material properties of the concrete are unknown but it can be considered as ordinary structural concrete. A contact type transducer (accelerometer, sensitivity 10 mV/m^2^, resonant frequency ≥50 kHz) was glued to the concrete surface for use as a reference sensor. The enclosure with the MEMS microphone board and the measurement microphone was mounted maintaining a distance of 20 mm to the concrete surface. There was no direct contact of the enclosure with the concrete surface. A steel ball (diameter 9 mm) on a thin rod was used as impact device. Several impacts were manually produced directly next to the array. All the array microphones, the accelerometer and the measurement microphone were located in a zone, where they could sense the ZGV-S_1_ Lamb mode generated by the impact.

**Figure 10:**
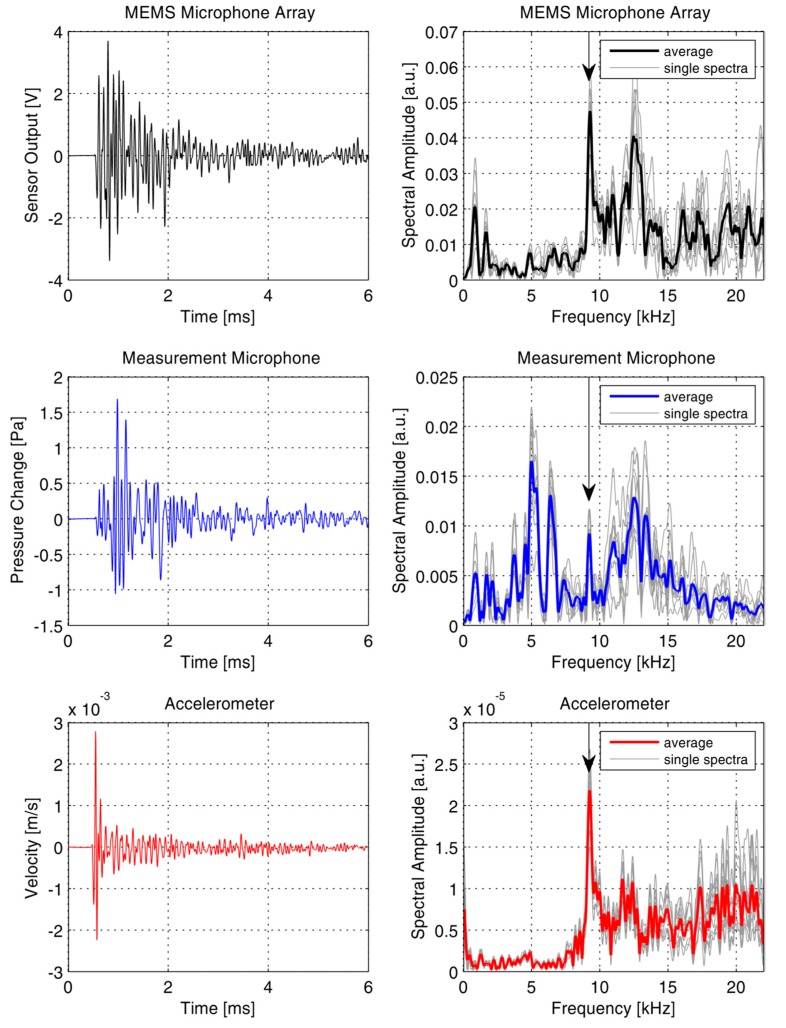
Raw data of a single impact (**Left Column**) and Fourier spectra (**Right Column**) of the Impact-Echo test measurement. The arrows indicate the frequency of the Impact-Echo resonance.

The microphones measure air pressure changes. These pressure changes are proportional to the out-of-plane velocity of the concrete surface [10,12]. Therefore, before further data analysis and sensor comparisons, the recordings from the accelerometer were numerically integrated to get the surface velocity. As in conventional IE processing, the recorded data was converted to the frequency domain by means of a Fourier transformation. Figure 10 (right column) shows the frequency spectra of ten single impacts and the average spectrum of these impacts. Records where taken with a sampling frequency of 200 kHz for 6 ms. The amplitude resolution of the digitizer was 16 bit. The Fourier spectra of the accelerometer data clearly show a single resonance peak. Assuming a typical P-wave velocity for structural concrete (≈4000 m/s), this peak at 9.3 kHz can be unambiguously identified as the IE thickness resonance (ZGV-S_1_ Lamb mode). This peak is also very pronounced in the recordings of the MEMS microphone array. In the recordings of the measurement microphone, no dominant single frequency peak can be easily identified. A peak at 9.3 kHz is visible but it is weaker than many other spectral peaks. These peaks are most likely the effect of direct impact noise, reverberations of the impact device and multiple reflections of such sound waves. The recorded data show that the MEMS microphone array has a much higher and more selective sensitivity to the acoustic waves originating from the concrete wall than the measurement microphone. In the MEMS microphone recordings, the peak at 9.3 kHz has the highest amplitude in the averaged spectrum and most of the single spectra.

**Figure 11:**
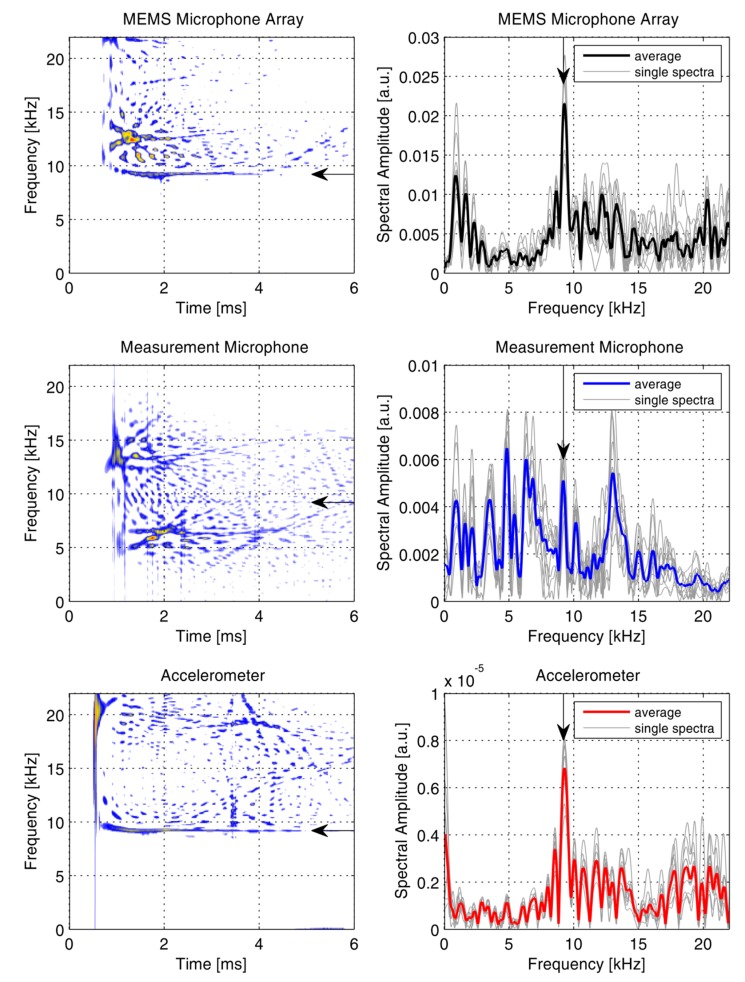
Time-frequency representation of a single impact (**Left Column**) and Fourier spectra of time windowed recordings (**Right Column**). The arrows indicate the frequency of the Impact-Echo resonance.

However, compared to the accelerometer data, the array recordings are not totally free from disturbances, most likely due to impact noise influences. For further analysis, a time-frequency representation of the data was produced by means of a Choi–Williams transformation (Figure 11, left column). The MEMS microphone array sensor and the accelerometer data clearly show high values at the IE resonance frequency (see arrows). In the recordings of the measurement microphone energy values at this frequency barely rise above the background level. As a further observation, it can be noted that the recordings of both microphone based sensors show pronounced high-energy values in the frequency range between 10 and 15 kHz. This energy is concentrated at time instants <2 ms and can be attributed to direct impact noise. Based on these observations, in the time-frequency representations a time window can be selected that separates the IE resonance peak from other energy and again a Fourier transformation is computed (Figure 11, right column). Only samples from a time window between 1.8 ms and 6 ms where passed to the Fourier transformation. Now the IE resonance peak is clearly the most prominent peak in the spectra of the array recordings. This is the case for the averaged spectra, but it also holds true for single impact measurements. Time windowing brings no improvement regarding the ambiguity in the spectral peaks of the measurement microphone.

The test measurement proved that the MEMS microphone array has the desired performance in terms of high sensitivity to the airwaves caused by the ZGV-S_1_ Lamb wave. Influences of direct impact noise are still present but much weaker compared to recordings of the single measurement microphone. This disturbance can easily be handled in the final data interpretation by simple time windowing. It should be noted that time windowing is often necessary even with contact sensor based IE measurements due to strong direct Rayleigh waves that can obscure the ZGV-S_1_ Lamb wave peak.

## 5. Conclusions/Outlook

We have shown by means of numerical simulation of stress wave propagation in a plate that the wave motions relevant for data interpretation in an IE test can be effectively measured by using several sensors distributed in a spatial array around the impact location. This is a significant modification to the conventional IE test configuration where the response of a structure to an impact source is measured at a single receiver location. Combining this new sensing concept with MEMS microphones led to the development of a prototype sensor board for air-coupled IE measurements. Comparison measurements of the developed MEMS microphone array with a precision measurement microphone and a conventional contact type transducer in a typical IE test setup have clearly shown the advantages of the new IE sensor.

By sensing the wave field in a spatial array, the overall sensor output has a high and selective sensitivity to the air-coupled ZGV-S_1_ Lamb mode. This particular mode has the greatest value for final data interpretation in the IE method and can be difficult to identify in single sensor recordings. No direct coupling of transducers is necessary with the array sensor. Even the direct contact of a sound insolating microphone housing can be avoided. Signal degradation due to bad sensor coupling and problems related to contact resonances or wear of sensor surfaces can be completely avoided.

As a further advantage, an array of microphones is less sensitive to unwanted acoustic signals such as background noise or direct impact noise than a single microphone. The mentioned benefits can be exploited by using the developed sensor in a scanning manner. In this way planar IE scans will become feasible with less effort. This will greatly enhance the reliability and informative value of the IE method.

Further studies will examine the suitability of the array sensing method for related elastic wave methods (e.g., spectral analysis of surface waves). By introducing phase shifts to the individual microphone outputs or by inclining the array, a focusing effect towards other wave types can be achieved.

For certain measurement tasks, a frequency calibration might be necessary since the frequency response of the MEMS microphones is not as flat as for a high quality measurement microphone. However, in conventional IE testing, only the correct identification of frequency peaks and their variations is relevant and not the corresponding true amplitude. Because of the low cost of MEMS microphones, as they are abundant components of many of today’s microelectronic devices like phones or laptops, the cost of MEMS based sensors is small compared to conventional contact type transducers used for IE.
